# Identification of a Devernalization Inducer by Chemical Screening Approaches in *Arabidopsis thaliana*

**DOI:** 10.3389/fpls.2021.634068

**Published:** 2021-02-04

**Authors:** Makoto Shirakawa, Yukaho Morisaki, Eng-Seng Gan, Ayato Sato, Toshiro Ito

**Affiliations:** ^1^Division of Biological Science, Graduate School of Science and Technology, Nara Institute of Science and Technology, Ikoma, Japan; ^2^Temasek Life Sciences Laboratory, National University of Singapore, Singapore, Singapore; ^3^Institute of Transformative Bio-Molecules (WPI-ITbM), Nagoya University, Nagoya, Japan

**Keywords:** vernalization, devernalization, chemical screening, *FLC*, epigenetics, H3K27me3, high-throughput screening, *Arabidopsis*

## Abstract

Vernalization is the promotion of flowering after prolonged exposure to cold. In *Arabidopsis thaliana*, vernalization induces epigenetic silencing of the floral repressor gene *FLOWERING LOCUS C* (*FLC*). The repressive epigenetic mark trimethylation of lysine 27 on histone H3 proteins (H3K27me3) is a critical contributor to the epigenetic silencing of *FLC*. Interestingly, the deposited H3K27me3 in the *FLC* locus can be erased by short-term high-temperature treatment. This is referred to as devernalization. In this study, we identified a novel chemical compound, 4-Isoxazolecarboxylic acid, 3,5-dimethyl-2-(4-fluorophenyl)-4-isoxazole carboxylic acid 1-methyl-2-oxoethyl ester named as DEVERNALIZER01 (DVR01), which induces devernalization in *Arabidopsis* seedlings, by an *FLC*-luciferase reporter-based high-throughput screening assay. DVR01 decreased the amount of H3K27me3 in the *FLC* locus in vernalized plants, resulting in the upregulation of *FLC* in the whole plant, including the vasculature and meristem, where FLC represses floral induction genes. We also showed that a 2-week treatment with DVR01 reverted plants with a vernalized status back to a fully non-vernalized status. Collectively, this study provides a novel structure of DVR01, which modulates devernalization via demethylation of H3K27me3 in the *FLC* locus.

## Introduction

Flowering is a critical developmental process in the plant life cycle. Many annual plants flower after being exposed to warm conditions following prolonged winter coldness (Chouard, 1960; Simpson and Dean, 2002). Acquiring the ability to undergo flower-bud formation induced by the cold is referred to as vernalization. In a model plant, *Arabidopsis thaliana*, flowering is promoted by both the vernalization pathway and autonomous pathway (Sheldon et al., 2000; Simpson and Dean, 2002; Michaels et al., 2005) and is inhibited by the activity of the zinc finger protein FRIGIDA (FRI) (Johanson et al., 2000). The vernalization pathway and autonomous pathway repress the expression levels of the floral repressor gene *FLOWERING LOCUS C* (*FLC*) (Michaels and Amasino, 1999; Sheldon et al., 1999, 2000; Simpson and Dean, 2002), and they counteract *FRI*, which activates the expression of *FLC* during the development of plants (Johanson et al., 2000). *FLC* acts upstream and represses the downstream floral inducers *FLOWERING LOCUS T* (*FT*) and *SUPPRESSOR OF OVEREXPRESSION OF CONSTANS 1* (*SOC1*) (Hepworth et al., 2002; Michaels et al., 2005; Helliwell et al., 2006; Searle et al., 2006). The vernalization pathway has a primary role in the regulation of *FLC* and triggers multiple repressive epigenetic modifications of the *FLC* locus, including the trimethylation of lysine 27 of histone H3 (H3K27me3) and H3K9me2 in a stepwise fashion (Bastow et al., 2004; Whittaker and Dean, 2017).

Interestingly, the vernalized state can be canceled by short-term treatment at a high temperature. This is referred to as devernalization (Purvis and Gregory, 1945; Gregory and Purvis, 1948). In the model plant *Arabidopsis thaliana*, the expression levels of *FLC* are partially recovered after devernalization (Périlleux et al., 2013). H3K27me3 is erased, and H3K4me3 very slightly accumulates at the *FLC* locus during devernalization (Bouché et al., 2015). These observations suggest that the decrease in H3K27me3 is a key event during devernalization. However, the detailed mechanisms of heat-induced devernalization are largely unknown. In this study, by employing a chemical biology approach, we aimed to identify novel chemicals that can induce devernalization in a model plant, *Arabidopsis thaliana*.

Chemical biology, in which researchers use small compounds to understand biological processes, has expanded in recent years (Nemhauser and Torii, 2016; Hagihara et al., 2019; Halder and Russinova, 2019). Plant researchers have identified compounds that mimic plant hormones (Tsuchiya et al., 2015; Hirakawa et al., 2017; Uchida et al., 2018; Yoshimura et al., 2018) and manipulate physiological processes, including membrane trafficking (Hicks and Raikhel, 2010; Dejonghe et al., 2019), circadian clock (Ono et al., 2019; Uehara et al., 2019), developmental patterning (Sakai et al., 2017; Ziadi et al., 2017), stomatal movement (Toh et al., 2018), and epigenetics (Sun et al., 2014). Chemical treatment can overcome genetic redundancy and transiently inhibit/activate protein function. To our knowledge, compounds regulating vernalization/devernalization have not yet been identified.

In this study, we performed screening by using the Institute of Transformative Bio-Molecules (ITbM) chemical library, which is one of most broadly used libraries in the plant science field (Toh et al., 2018; Yoshimura et al., 2018; Ono et al., 2019; Uehara et al., 2019). We established a luciferase-based high-throughput screening system. From 3010 compounds, we identified a novel compound, DEVERNALIZER01 (DVR01), which activates the expression of the floral master repressor *FLC*, in vernalized plants. In addition, DVR01-treated vernalized plants exhibited a late flowering phenotype. These results suggested that DVR01 is an inducer of devernalization in *Arabidopsis*. Finally, we showed that DVR01 decreased the accumulation levels of a repressive histone mark H3K27me3, at the *FLC* locus. This study provides a novel structure of devernalization-inducing compounds. DVR01 may be useful for the induction of devernalization instead of heat treatment in agriculture.

## Materials and Methods

### Plant Materials and Growth Conditions

All *Arabidopsis thaliana* seed stocks used in this study were in the Columbia (Col-0) background. *flc-3* (Michaels and Amasino, 1999), *FRI*^*sf*–2^ (Lee et al., 1993) and the reporter line *FLC-GUS* (Bastow et al., 2004) were reported previously. *FLC:LUC* was generated in this study. *Arabidopsis* seeds were grown on cotton balls with liquid Murashige Skoog (MS) medium or on 0.5% gellan gum with MS. Plates were cultivated under constant light conditions. To examine the flowering phenotypes, plants were cultivated in pots containing vermiculite and Metro-Mix (Sun Gro Horticulture).

### Plasmid Construction and Plant Transformation

A chimeric gene between *FLC* and *luciferase* was named *FLC:LUC*. The luciferase gene was fused to the 6th exon of *FLC*. The 7th exon of *FLC* is not translated because the luciferase gene has a stop codon. Therefore, FLC:LUC does not have the activity of FLC. This DNA construct was introduced into the *flc-3 FRI*^*sf*–2^ background. We selected T3 transgenic lines harboring one copy of the construct and checked the luciferase activity in both non-vernalized conditions and vernalized conditions.

### Luciferase Assay

We vernalized *FLC:LUC* seeds after water absorption in microtubes in a refrigerator (4 degrees). After 4 weeks of vernalization, we sowed four seeds on cotton balls with MS medium containing compounds (Toh et al., 2018; Yoshimura et al., 2018; Ono et al., 2019; Uehara et al., 2019) at 10 μM in 96-well black plates. Seven-day-old seedlings were sprayed with 1 mM D-Luciferin (Sigma) in 0.01% Triton X-100. We measured luciferase activities by ImageQuant^TM^ LAS 4000 (GE Healthcare). Images and signals are the sum of LUC activity over 10 min.

### Reverse-Transcription PCR and Quantitative RT-PCR

After 4 weeks of vernalization, we sowed four seeds on 0.5% gellan gum with MS and 1, 5, 10, and 25 μM DVR01. Samples of 7-day-old seedlings were frozen in liquid nitrogen immediately. The RNeasy Plant Mini Kit (Qiagen, Germany) was used to extract total RNA. The RNase-Free DNase Set (Qiagen, Germany) was used to eliminate the contamination of genomic DNA in RNA samples. Reverse-transcription PCR was performed using PrimeScript^TM^ RT Master Mix (Takara, Japan). Quantitative RT-PCR was applied as described previously (Wang et al., 2020). *Arabidopsis PP2A* was used as the internal reference. Each experiment was repeated at least three times. The relative expression level of each gene was calculated using the 2^–ΔΔ*CT*^ method (Livak and Schmittgen, 2001). Primers are listed in Supplementary Table 1.

### GUS Staining

After 4 weeks of vernalization, we sowed four seeds on 0.5% gellan gum with MS and 10 μM DVR01. Seven-day-old seedlings of *FLC-GUS* were fixed in 90% acetone for 30 min at room temperature and subsequently stained with GUS staining solution. The staining method was described previously (Shirakawa et al., 2014). Representative images were photographed under an AXIO Zoom V16 (ZEISS) microscope.

### Flowering Phenotype Analysis

To test the timing of flowering, including the number of rosette or cauline leaves produced, we cultured seedlings for one or 2 week(s) in 10 μM DVR01-containing medium after 4 weeks of vernalization and then transferred them into soil cultivation conditions. We cultivated plants until the length of the primary stems reached 10 cm and then counted the number of leaves.

### Chromatin Immunoprecipitation (ChIP)-qPCR

For ChIP-qPCR, ChIP was carried out as described previously (Yamaguchi et al., 2014). After 4 weeks of vernalization, we sowed four seeds on 0.5% gellan gum with MS and 10 μM DVR01. Samples of 7-day-old seedlings were collected. For each sample, 100–300 mg of seedlings was fixed with 1% formaldehyde for 15 min. After quenching the formaldehyde with glycine for 5 min, tissues were frozen in liquid nitrogen and kept at −80°C until use. Tissues were ground to a fine powder with an ice-cold mortar and pestle. Using nuclear extraction buffer, chromatin was isolated from a nuclear extract. Fragmentation was conducted using an Ultrasonic Disruptors UD-201 sonicator (TOMY). After preclearing, antibodies were added, and the mixtures were rotated overnight at 4°C. Antibodies, anti-H3K27me3 (ab6002; Abcam), were used. For immunoprecipitation to capture DNA-protein complexes, Dynabeads with Protein A or G (Thermo Fisher Scientific) were used. Beads were washed, and DNA was eluted from beads overnight at 65°C. The resulting DNA was purified using a QIAquick PCR Purification Kit (Qiagen). DNA was quantified with a LightCycler 480 (Roche) using FastStart Essential DNA Green Master Mix (Roche). The ratio of ChIP to input DNA (% input) was compared based on the reaction threshold cycle for each ChIP sample compared to a dilution series of the corresponding input sample. Relative values are normalized by the negative control locus of the TA3 retrotransposon (At1g37110) (Yamaguchi et al., 2014). Three independent experiments were performed. Primers are listed in Supplementary Table 1.

### Data Statistics and Availability

In this study, one-way ANOVA followed by the Tukey–Kramer test or two-tailed Student’s *t*-test with Bonferroni correction for multiple comparisons was performed to detect differences as required.

## Results

### *Luciferase*-Based High-Throughput Screening System for Monitoring the Expression of *FLC*

**FIGURE 1:**
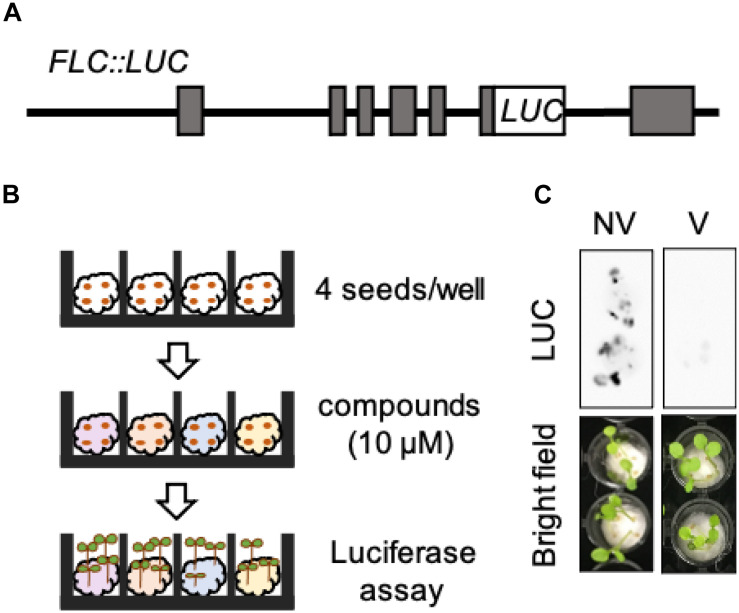
*Luciferase*-based high-throughput screening system for monitoring the expression of *FLC*. **(A)** Structure of the *FLC:LUC* DNA construct. **(B)** Schematic diagram of luciferase assay. **(C)** Luciferase activities of *FLC:LUC* in NV and V plants. Upper images are original photographs taken by LAS 4000. Lower images are bright field images.

To perform high-throughput screening for novel chemicals inducing devernalization, we established the *FLC:luciferase* reporter line (hereafter, *FLC:LUC*) (Figure 1A). We introduced a chimeric gene of *FLC*, in which the luciferase gene was fused to the 6th exon of *FLC*, into the *flc-3 FRI*^*sf*–2^ background. This construct contains all known *cis-*regulatory sequences of *FLC*. In addition, compared with *fri*^*Col*–0^, *FRI*^*sf*–2^ can fully activate *FLC*. Therefore, plants harboring *FRI*^*sf*–2^ exhibited a clear reduction in *FLC* in response to vernalization treatment. In this study, we represented vernalized plants as V plants and non-vernalized plants as NV plants, respectively. We selected a single-copy transgenic line with *FLC:LUC* expression similar to endogenous *FLC*. In this line, cold treatment of seeds for 4 weeks after water absorption induced a clear and significant reduction in luciferase activity in the seedlings (Figures 1B,C). These results indicate that we are able to monitor the dynamic expression levels of *FLC* in a non-destructive manner by measuring LUC activity in *FLC:LUC*.

### Identification of a Hit Compound, DVR01

**FIGURE 2:**
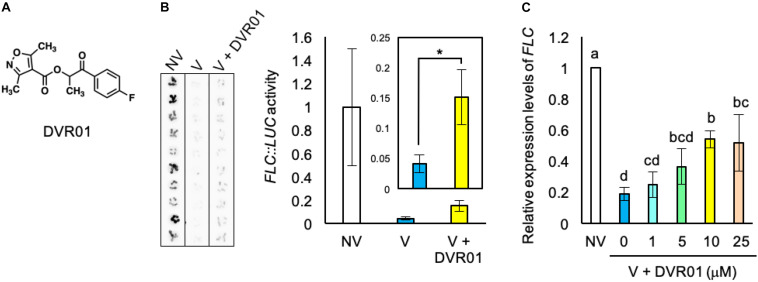
Identification of a devernalization inducer, DVR01. **(A)** Chemical structure of DVR01. **(B)** Luciferase activities of *FLC:LUC* in NV-, V-, and DVR01-treated V plants. DVR01 at 10 μM was used. Left photographs are original images taken by LAS 4000. Right graphs indicate quantified values of LUC activity from original images. Error bars represent SD. Two-tailed Student’s *t*-test with Bonferroni correction. *p < 0.05. **(C)** The expression levels of endogenous *FLC* in NV-, V-, and DVR01-treated V plants. DVR01 at 1, 5, 10, and 25 μM was used. Error bars represent SD. One-way ANOVA followed by the Tukey–Kramer test was performed (*p* < 0.05). Different letters indicate significant differences, while the same letters indicate non-significant differences.

We searched for synthetic small molecules that could activate the expression of *FLC* in V plants from the ITbM chemical library, our unique chemical library for use in plant-based phenotypic screening (Toh et al., 2018; Yoshimura et al., 2018; Ono et al., 2019; Uehara et al., 2019). We screened 3010 molecules and found two compounds that moderately recovered *LUC* activity in V plants. In this study, we show the results of hit compound, DEVERNALIZER01 (DVR01) (Figure 2A), and will report the other molecule in future studies. DVR01-treated V plants showed 3.2-fold higher luciferase activity than mock-treated V plants (Figure 2B; *p* < 0.05, two-tailed Student’s *t*-test with Bonferroni correction). To avoid the possibility that DVR01 activated the enzymatic activity of LUC, we measured the expression levels of endogenous *FLC* by using *FRI*^*sf*–2^ plants. Consistent with the experiments using *FLC:LUC*, DVR01-treated V plants showed 2.9-fold higher expression levels of endogenous *FLC* than V plants (Figure 2C; *p* < 0.05, Tukey–Kramer test). In addition, the expression level of *FLC* in DVR01-treated NV was comparable with that in NV plants (Supplementary Figure 1). These results suggested that DVR01 induced the expression of *FLC* specifically in V plants. To find the optimum and effective concentration of DVR01, we treated V plants with DVR01 at concentrations of 1, 5, 10, and 25 μM DVR01 and identified that both 10 and 25 μM DVR01 induced the expression of *FLC* at maximum levels (Figure 2C; *p* < 0.05, Tukey–Kramer test). On the other hand, we found DVR01 induced undesired side effects, dwarfism of plants and pale green leaves, in a concentration-dependent manner. At a concentration of 25 μM of DVR01, plants showed severe dwarfism (Supplementary Figure 2). Therefore, in this study, we used 10 μM DVR01 in *Arabidopsis*. Next, to clarify a relationship between structure and activity of DVR01, we selected 30 structurally related compounds having 4-isoxazolecarboxylic acid moiety from the chemical library and tested them (Supplementary Figure 3). However, no compound induced *FLC* expression, dwarfism or pale green leaves. These results suggested that the whole structure of DVR01 may be privileged scaffold for its activity (see section “Discussion”).

### DVR01 Upregulates the Expression of *FLC* in Leaves of V Plants

**FIGURE 3:**
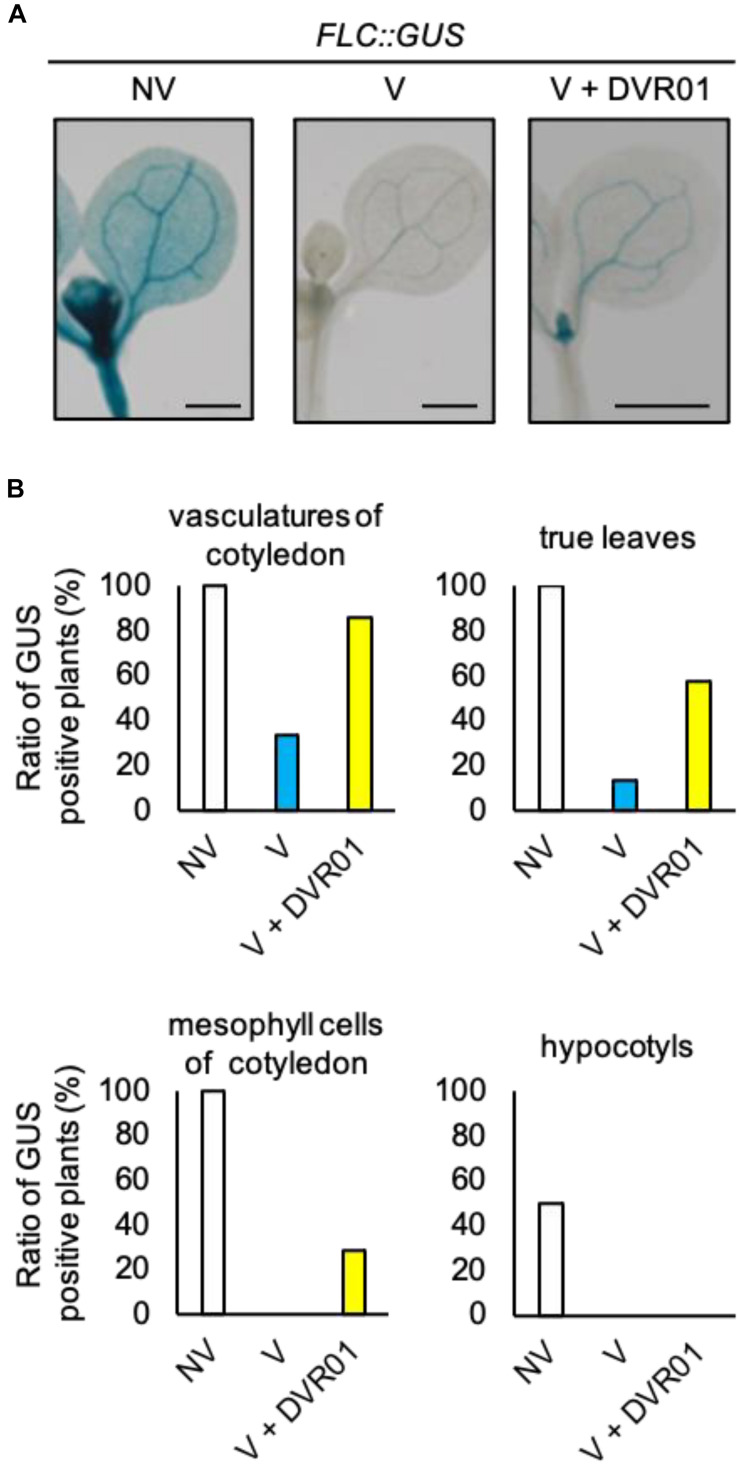
DVR01 upregulates the expression of *FLC* in the leaves of V plants. **(A)** Photographs of GUS staining of NV-, V-, and DVR01-treated *FLC:GUS*, including cotyledons, true leaves and hypocotyls. Bars are 500 μm. **(B)** Ratio of GUS-positive plants in different tissues. NV; *n* = 10, V; *n* = 15, V + DVR01; *n* = 14.

*FLOWERING LOCUS C* is expressed in whole plant tissues, including aerial parts and roots. However, the expression of *FLC* only in vascular and meristematic tissues of aerial parts is critical for the repression of *FT*. To examine the tissues in which DVR01 induces *FLC*, we treated the translational fusion line *FLC-GUS* with 10 μM DVR01 after vernalization and stained whole seedlings. We found much stronger GUS signals in both vascular tissues of cotyledons and true leaves of DVR01-treated V plants compared with those of vernalized *FLC-GUS* plants (Figure 3A). Eighty-six percent of DVR01-treated V plants had GUS signals in vascular tissues of cotyledons, compared with 33% of V plants (Figure 3B, DVR01-treated V plants, *n* = 14; V plants, *n* = 15). In addition, 57% of DVR01-treated V plants had GUS signals in true leaves compared with 13% of V plants (Figure 3B, DVR01-treated V plants, *n* = 14; V plants, *n* = 15). Meanwhile, no hypocotyl was stained by GUS in either V or DVR01-treated V plants (Figure 3B). These results indicate that DVR01 induced the reactivation of *FLC*, especially in leaves, resulting in the repression of *FT* in those tissues.

### DVR01 Induces Devernalization

**FIGURE 4:**
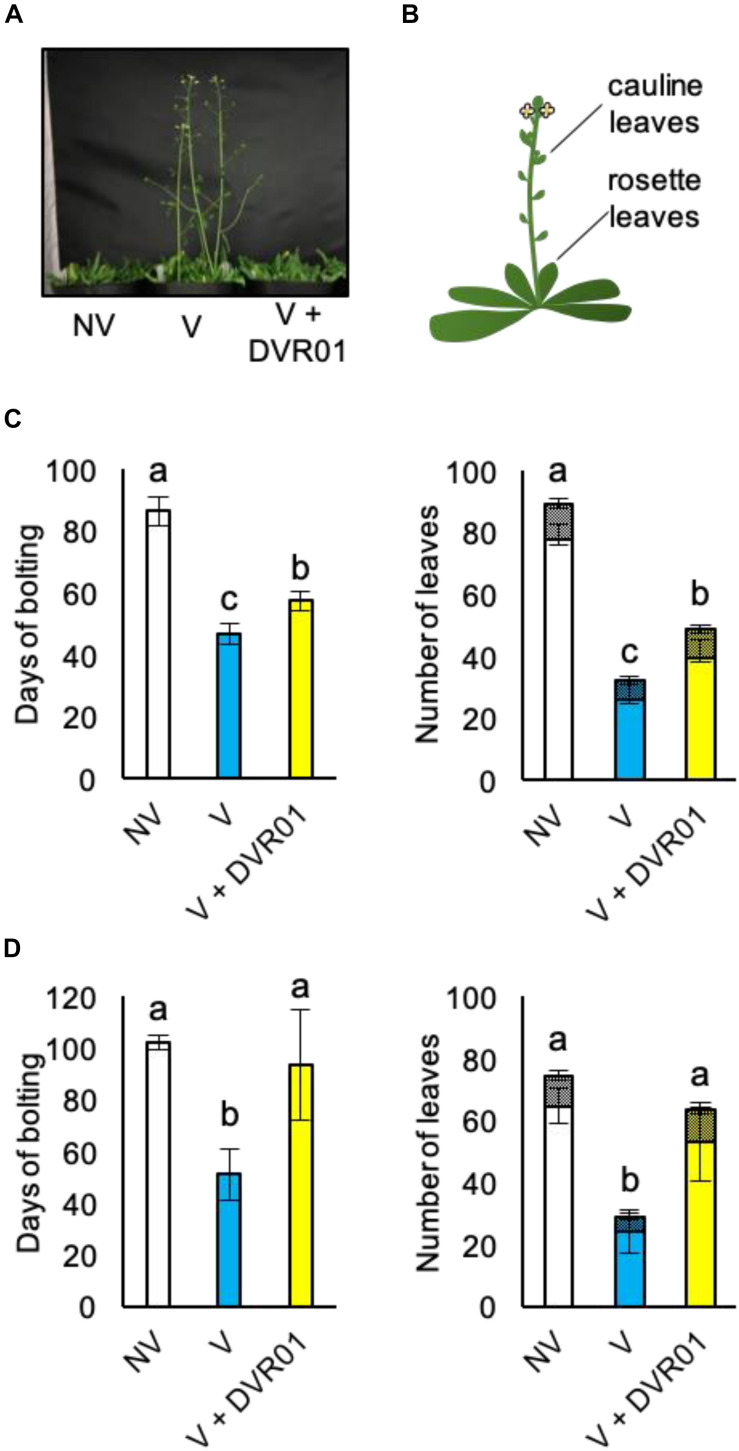
DVR01 induces devernalization. **(A)** A photograph of 49-day-old plants. **(B)** Schematic diagram of two types of leaves counted in the flowering assay. **(C)** One week of treatment with DVR01 in V plants delayed the timing of flowering. **Left:** days of bolting. **Right:** number of leaves including cauline (hatched boxes) and rosette leaves (box). NV: *n* = 7, V: *n* = 10, V + DVR01: *n* = 8. Error bars represent SD. One-way ANOVA followed by the Tukey–Kramer test was performed (*p* < 0.05). Different letters indicate significant differences, while the same letters indicate non-significant differences. **(D)** Two weeks of treatment with DVR01 in V plants delayed the timing of flowering. **Left:** days of bolting. **Right:** number of leaves including cauline (hatched boxes) and rosette leaves (box). NV: *n* = 6, V: *n* = 5, V + DVR01: *n* = 5. Error bars represent SD. One-way ANOVA followed by the Tukey–Kramer test was performed (*p* < 0.05). Different letters indicate significant differences, while the same letters indicate non-significant differences.

Analyses of the expression profiles of *FLC* in DVR01-treated V plants suggested that DVR01 could confer late-flowering phenotypes against vernalized plants. To investigate this possibility, we cultured seedlings for 1 week in 10 μM DVR01-containing medium; after that, we transferred them into soil conditions, and we counted both the day of bolting and the numbers of rosette and cauline leaves (Figures 4A,B). DVR01-treated V plants showed late-flowering phenotypes compared with untreated V plants (Figure 4C). DVR01-treated V plants bolted 11 days later than V plants (Figure 4C; 57 days for DVR01-treated V plants vs. 46 days for V plants; *p* < 0.05, Tukey–Kramer test) and increased vegetative growth relative to V plants (Figure 4C; DVR01-treated V plants developed 17 more leaves than V plants did; *p* < 0.05, Tukey–Kramer test). Next, we treated V plants for 2 weeks with 10 μM DVR01. The effects of DVR01 were enhanced (Figure 4D). DVR01-treated V plants bolted at almost the same time as non-vernalized plants (no significant difference by Tukey–Kramer test) and developed almost the same number of leaves (no significant difference by Tukey–Kramer test) (Figure 4D). These results indicated that DVR01 could revert plants to a non-vernalized status. Collectively, these results suggest that DVR01 is a novel compound that promotes devernalization in plants.

### Low Levels of H3K27me3 Accumulation at the *FLC* Locus in DVR01-Treated V Plants

**FIGURE 5:**
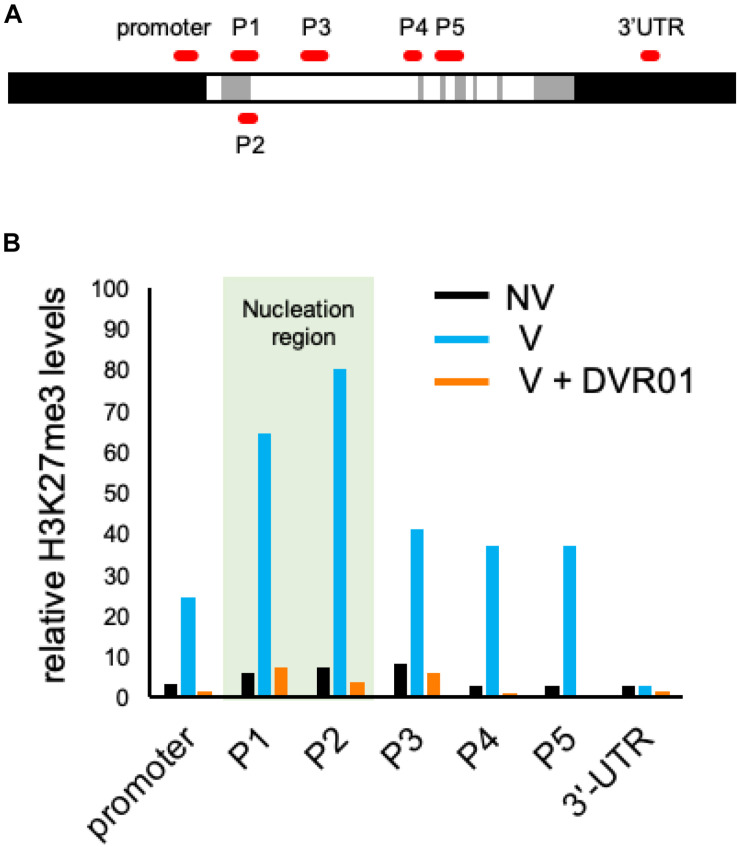
Low accumulation levels of H3K27me3 on the *FLC* locus in DVR01-treated V plants. **(A)** Sites of PCR amplicons on the *FLC* locus. **(B)** The accumulation levels of H3K27me3 in NV-, V-, and DVR01-treated V plants. The P1 and P2 regions are located in the nucleation region (light green). Additional replicates are shown in Supplementary Figure 4. Relative values are normalized by the negative control TA3.

Vernalization induced the accumulation of the repressive epigenetic mark H3K27me3 at the whole *FLC* locus. The accumulation of H3K27me3 is first introduced at the region of the 1st intron of the *FLC* locus and continuously spread throughout the whole genomic region of *FLC* except the 3′-UTR (Whittaker and Dean, 2017). We compared the accumulation levels of H3K27me3 in the entire *FLC* genomic region between NV-, V-, and DVR01-treated V plants by using ChIP-qPCR with three biological replicates (Figure 5 and Supplementary Figure 4). Compared with NV plants, V plants exhibited higher accumulation levels of H3K27me3 on the whole *FLC* locus except the 3′-UTR. Higher accumulation levels were detected in the P1 and P2 regions than in the promoter and P3–P5 regions. These results were consistent with previous reports (Whittaker and Dean, 2017). Compared with V plants, DVR01-treated V plants showed lower accumulation of H3K27me3 in the whole *FLC* locus except the 3′-UTR, similar to NV plants (Figure 5B). These results were reproduced in two additional independent experiments (Supplementary Figure 4). These findings indicate that DVR01 eliminates H3K27me3 from the *FLC* locus. Taken together, DVR01 is a novel compound that induces a late-flowering phenotype against V plants through the reactivation of *FLC* by reducing the repressive epigenetic marks from the *FLC* locus.

## Discussion

### Identification of a Devernalization Inducer, DVR01

In this study, we found that a novel compound, DVR01, has the ability to induce the expression of *FLC* in leaves and delay flowering in vernalized plants (Figures 2–4). DVR01 does not share the clear structural similarity with plant hormones or known H3K27me3 inhibitors. The activity of DVR01 was dose-dependent, but a high concentration of DVR01 retarded plant growth (Figure 2C and Supplementary Figure 2). Instead of using a high concentration of DVR01, longer treatment periods of DVR01 permitted us to revert vernalized status back to the non-vernalized status almost perfectly (Figure 4D). Then, to understand structure-activity relationship of DVR01, we selected 30 structurally related compounds having 4-isoxazolecarboxylic acid moiety and tested them as with the same manner mentioned above. Unfortunately, none of the compounds induced *FLC* expression or morphological changes, as DVR01 did (Supplementary Figure 3). These results suggested that the whole structure of DVR01 may be privileged scaffold for its activity.

Our study also revealed that DVR01 could manipulate the histone modification H3K27me3 of the *FLC* locus by ChIP-qPCR analysis (Figure 5). Consistent with this, DVR01 did not upregulate the expression of *FLC* in NV plants (Supplementary Figure 1), in which H3K27me3 was not deposited on the *FLC* locus (Figure 5; Whittaker and Dean, 2017). How specific DVR01 affects the accumulation levels of H3K27me3 on the *FLC* locus? In addition to the upregulation of *FLC*, DVR01-treated plants showed the dwarfism and the pale green leaves. It might be caused by the loss of H3K27me3 on the locus of developmental regulators which inhibit the proper growth and maturation of chloroplasts when they are ectopically expressed at inappropriate tissues/cells. However, phenotypes of DVR01-treated plants were not identical with those of mutants of PRC2 complex (for example, *CURLY LEAF*). This result suggests that DVR01 has some specific activity against H3K27me3. Future research employing ChIP-seq may serve to elucidate in detail the mechanisms of DVR01 in the accumulation of H3K27me3.

In the future, further structure-activity relationship study of DVR01 may reveal a privileged scaffold to identify target molecule(s) of DVR01, which will provide more detailed mechanistic insights into DVR01 in plants. Additionally, our high-throughput screening system will identify other molecules that have devernalization-inducing activity without harmful effects on plant growth and clarify common structures for devernalization-inducing compounds.

### Heat Versus Chemicals in Devernalization

It was reported in the 1940s that heat could cancel vernalized status in plants (Purvis and Gregory, 1945; Gregory and Purvis, 1948). In this study, we showed that a small molecule, DVR01, could also induce devernalization in vernalized seeds of *Arabidopsis*. It remains an open question whether DVR01 functions by similar mechanisms to heat in devernalization. In *Arabidopsis*, it was reported that heat-treated V plants showed about 2- to 3-fold higher expression levels of *FLC* than V plants (Périlleux et al., 2013). Compared with this, DVR01 increased the expression levels of *FLC* to a similar extent against V plants (Figure 2C). However, more detailed work, including omics analyses (RNA-seq and ChIP-seq) and the combinational treatment of heat and chemicals, is required for the comparison of two kinds of devernalization.

In our study, we also found that heat treatment of seeds under dark conditions followed by cultivation under light conditions was required for the effective induction of devernalization (Shirakawa and Ito, unpublished data). In addition, in *Arabidopsis*, 1 week of heat treatment of seeds in the dark triggered a clear reduction in the germination ratio of seeds (Shirakawa and Ito, unpublished data). Moreover, DVR01 treatment was performed under light conditions during 1 week of cultivation after the vernalization of seeds. Therefore, it is not easy to directly compare heat-induced devernalization and DVR01-induced devernalization. However, we did not exclude the possibility that targets of heat treatment may overlap with those of DVR01 treatment because they reactivated the expression levels of *FLC* through a decrease in H3K27me3 at the *FLC* locus (Figure 5; Périlleux et al., 2013; Bouché et al., 2015).

### Chemicals for the Modification of H3K27me3 in Plants

We showed that DVR01 could revert the epigenetic status determined by H3K27me3 on the *FLC* locus. How and when does DVR01 function in the regulation of H3K27me3? First scenario is that H3K27me3 spreading is established in seeds just after cold treatment and DVR01 might actively exclude the H3K27me3 through histone demethylases. However, it is still unclear when H3K27me3 spreading and stable silencing of *FLC* are established only upon exposure to warmth (Whittaker and Dean, 2017). Second scenario is that H3K27me3 spreading is established during the development of seedling from the germination and DVR01 might inhibit the activity of PRC2 and/or activate histone demethylases to counteract PRC2. Recently, it was proposed that DNA replication may have an active role in propagating repressive histone marks (Whittaker and Dean, 2017). Therefore, DVR01 might also inhibits the spreading of H3K27me3 by affecting DNA replication. In addition to H3K27me3, other histone modifications, including H3K36me3 and H3K4me3, are involved in the regulation of *FLC* (Whittaker and Dean, 2017). It remains open question whether DVR01 function in the accumulation/deposition of other histone modifications. Future work may attempt to identify targets of DVR01. Moreover, using chemical compounds to regulate devernalization will shed light on the detailed mechanism of epigenetically stable and heritable silencing of *FLC*.

## Data Availability Statement

The original contributions presented in the study are included in the article/Supplementary Material, further inquiries can be directed to the corresponding authors.

## Author Contributions

MS, AS, and TI conceived this study and wrote the manuscript. MS and YM performed all the experiments except generating *FLC:LUC* transgenic plants. E-SG established *FLC:LUC* transgenic plants. All authors read and approved the final version of the manuscript.

## Conflict of Interest

The authors declare that the research was conducted in the absence of any commercial or financial relationships that could be construed as a potential conflict of interest.

## Supplementary Material

Supplementary Figure 1 DVR01 did not affect the expression levels of *FLC* in NV plants. The expression levels of *FLC* were quantified in 7-day-old seedlings of NV plants and DVR01-treated NV plants. No statistical significance was detected. Error bars denote SD. Two-tailed Student’s *t*-test was performed.

Supplementary Figure 2 Plant morphologies of DVR01-treated plants. Photographs of 7-day-old seedlings treated with various concentrations of DVR01. DVR01 induced retarded growth in a concentration-dependent manner.

Supplementary Figure 3 *FLC:LUC* activities in V plants treated with analogous compounds of DVR01. **(A)** Chemical structures of 30 analogous compounds of DVR01. **(B)** *FLC:LUC* activities were measured in V plants treated with 30 analogous compounds of DVR01. Compounds at 10 μM were used. Compared with these plants, DVR01-treated V plants showed the highest activity of *LUC*. Error bars represent SD.

Supplementary Figure 4 Three replicates of ChIP-qPCR analysis of H3K27me3. The accumulation levels of H3K27me3 in NV-, V-, and DVR01-treated V plants. The P1 and P2 regions are located in the nucleation region (light green). Relative values are normalized by the negative control TA3.

Supplementary Table 1 Primers used in this study.
