# Supplementary material for: Identification of a Devernalization Inducer by Chemical Screening Approaches in Arabidopsis thaliana
Source: Front Plant Sci. 2021 Feb 4;12:634068. doi: 10.3389/fpls.2021.634068 (PMC7890032; doi:10.3389/fpls.2021.634068)
Supplement: Supplementary Figure 1 — DVR01 did not affect the expression levels of FLC in NV plants. The expression levels of FLC were quantified in 7-day-old seedlings of NV plants and DVR01-treated NV plants. No statistical significance was detected. Error bars denote SD. Two-tailed Student’s t-test was performed. [file Data_Sheet_1.pdf]

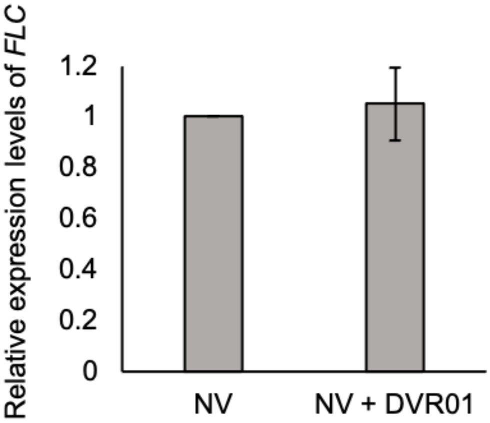

Supplementary Figure 1 | DVR01 did not affect the expression levels of *FLC* in NV plants. The expression levels of *FLC* were quantified in 7-day-old seedlings of NV plants and DVR01-treated NV plants. No statistical significance was detected. Error bars denote SD. Two-tailed Student's t-test was performed.

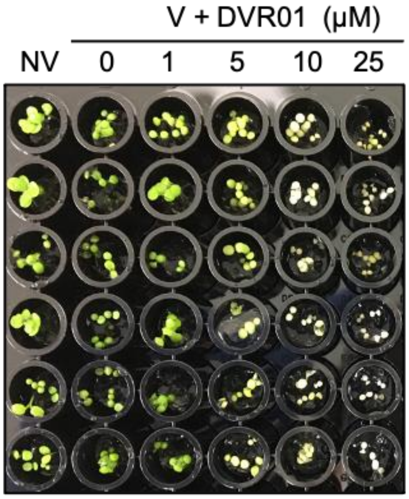

Supplementary Figure 2 | Plant morphologies of DVR01-treated plants. Photographs of 7-day-old seedlings treated with various concentrations of DVR01. DVR01 induced retarded growth in a concentration-dependent manner.

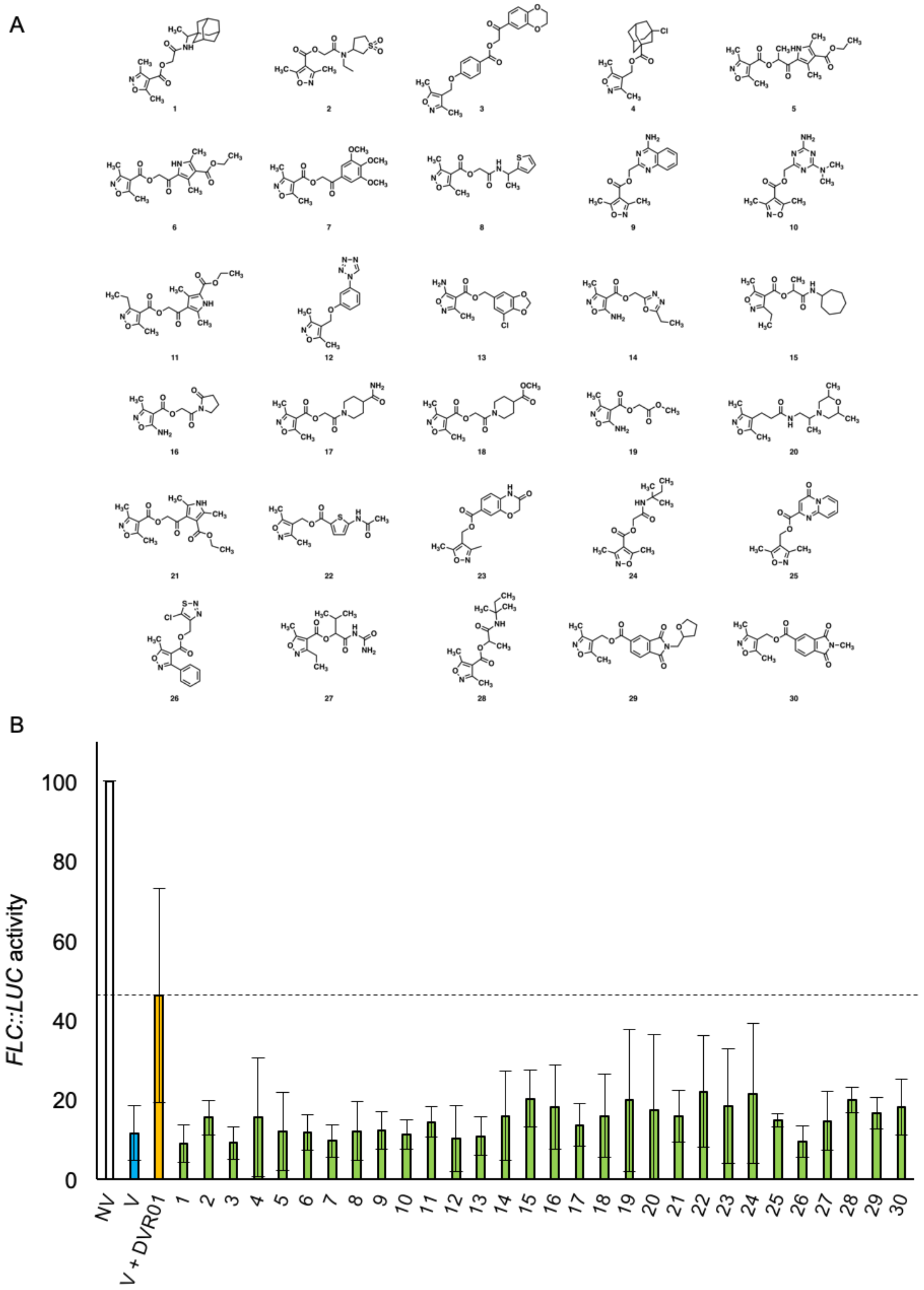

Supplementary Figure 3 | *FLC::LUC* activities in V plants treated with analogous compounds of DVR01. (A) Chemical structures of 30 analogous compounds of DVR01. (B) *FLC::LUC* activities were measured in V plants treated with 30 analogous

compounds of DVR01. Compounds at 10 μM were used. Compared with these plants, DVR01-treated V plants showed the highest activity of *LUC*. Error bars represent SD.

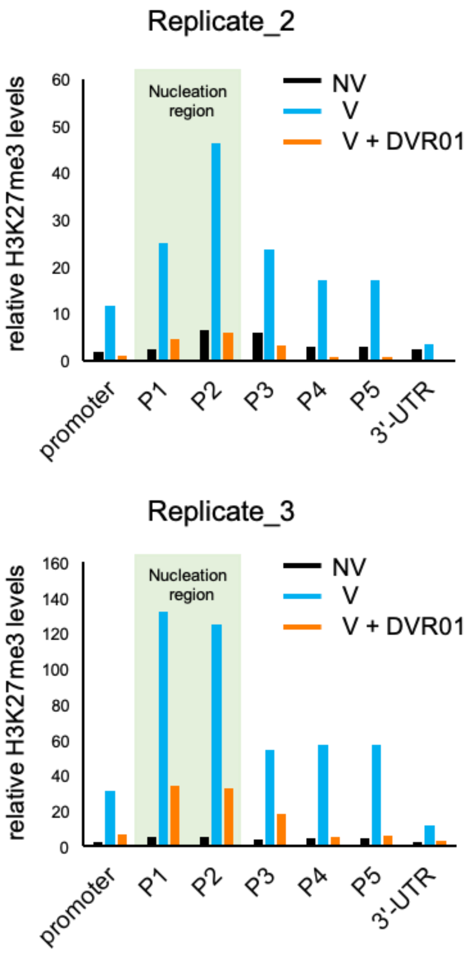

Supplementary Figure 4 | Three replicates of ChIP-qPCR analysis of H3K27me3. The accumulation levels of H3K27me3 in NV-, V-, and DVR01-treated V plants. The P1 and P2 regions are located in the nucleation region (light green). Relative values are normalized by the negative control TA3.

Supplementary Table 1 | Primers used in this study

Primers for qPCR	Sequence
PP2A (Fw)	5'-TATCGGATGACGATTCTTCGTGCAG-3'
PP2A (Rv)	5'-GCTTGGTCGACTATCGGAATGAGAG-3'
FLC (Fw)	5'-CCGAACTCATGTTGAAGCTTGTTGAG-3'
FLC (Rv)	5'-CGGAGATTTGTCCAGCAGGTG-3'

Primers for ChIP-qPCR	Sequence
FLC-promoter (Fw)	5'-ACTATGTAGGCACGACTTTGGTAAC-3'
FLC-promoter (Rv)	5'-TGCAGAAAGAACCTCCACTCTAC-3'
FLC-p1 (Fw)	5'-CGACAAGTCACCTTCTCCAAA-3'
FLC-p1 (Rv)	5'-AGGGGGAACAAATGAAAACC-3'
FLC-p2 (Fw)	5'-GTCGCTCTTCTCGTCGTCTC-3'
FLC-p2 (Rv)	5'-AGGGGGAACAAATGAAAACC-3'
FLC-p3 (Fw)	5'-TTCCTATCTTTGCTGTGGACCT-3'
FLC-p3 (Rv)	5'-GAATCGCAATCGATAACCAGA-3'
FLC-p4 (Fw)	5'-GTTTCCAGTGGCCTTTTCAA-3'
FLC-p4 (Rv)	5'-GACCAGGCTGGAGAGATGAC-3'
FLC-p5 (Fw)	5'-CTTTTTCATGGGCAGGATCA-3'
FLC-p5 (Rv)	5'-TGACATTTGATCCCACAAGC-3'
FLC-3'UTR (Fw)	5'-TTGTAAAGTCCGATGGAGACG-3'
FLC-3'UTR (Rv)	5'-ACTCGGCGAGAAAGTTTGTG-3'
TA3 (Fw)	5'-CTGCGTGGAAGTCTGTCAAA-3'
TA3 (Rv)	5'-CTATGCCACAGGGCAGTTTT-3'
